# Complete Chloroplast Genomes and Comparative Analyses of Three Ornamental Impatiens Species

**DOI:** 10.3389/fgene.2022.816123

**Published:** 2022-03-30

**Authors:** Chao Luo, Wulue Huang, Huseyin Yer, Troy Kamuda, Xinyi Li, Yang Li, Yuhong Rong, Bo Yan, Yonghui Wen, Qiong Wang, Meijuan Huang, Haiquan Huang

**Affiliations:** ^1^ College of Landscape Architecture and Horticulture Sciences, Southwest Research Center for Engineering Technology of Landscape Architecture (State Forestry and Grassland Administration), Southwest Forestry University, Kunming, China; ^2^ Yunnan Engineering Research Center for Functional Flower Resources and Industrialization, Southwest Forestry University, Kunming, China; ^3^ Research and Development Center of Landscape Plants and Horticulture Flowers, Southwest Forestry University, Kunming, China; ^4^ Department of Landscape Architecture and Plant Science, University of Connecticut, Storrs, CT, United States; ^5^ Faculty of Forestry, Duzce University, Duzce, Turkey

**Keywords:** *Impatiens*, *Balsaminaceae*, chloroplast genome, comparative analysis, phylogenetic relationship

## Abstract

*Impatiens* L., the largest genus in the family *Balsaminaceae* with approximately 1,000 species, is a controversial genus. Due to the conflict of morphological features and insufficient genomic resources, the studies of systematic evolution and understanding of taxonomic identification are considered to be very limited. Hence, we have sequenced the complete chloroplast genomes of three ornamental species (*Impatiens balsamina, I. hawkeri,* and *I. walleriana*), and compared them with previously published wild species data. We performed a detailed comparison of a highly similar basic structure, size, GC content, gene number, order, and functional array among them. Similarly, most divergent genes were detected from previous work in the literature. The mutational regions containing highly variable nucleotide hotspots were identified and may be used as potential markers for species identification and taxonomy. Furthermore, using whole chloroplast genome data to analysis the phylogenetic relationship of the *Balsaminaceae* species, we found that they were all part of a single clade. The three phenotypically different ornamental species were clustered together, suggesting that they were very likely to be closely related. We achieved and characterized the plastid genome structure, identified the divergence hotspots, and determined the phylogenetic and taxonomic positions of the three cultivated species in the Impatiens genus. The results may show that the chloroplast genome can be used to solve phylogenetic problems in or between the Impatiens genus and also provide genomic resources for the study of the Balsaminaceae family’s systematics and evolution.

## Introduction

The *Balsaminaceae* family consists of only two genera; the species-rich *Impatiens* L. and the monospecific *Hydrocera triflora* with substantial similarity in morphology and molecular biology datasets (Chen, 2001; Janssens et al., 2012)*.* The controversial and complex flowering genus *Impatiens*, consists of approximately 1,000 species, which are distributed from the tropics to subtropics and extend to temperate regions of tropical Africa, Southwest Asia, Southern China, Europe, Russia, and North America (Grey-Wilson, 1989; Yu, 2012). Tropical Africa, Madagascar, Sri Lanka, Himalayas, and Southeast Asian are the five biodiversity hotspots for the endemic *Impatiens* (Grey-Wilson, 1980; Chen, 2001). Due to the diverse flowering and morphological variables, many cultivars (*Impatiens balsamina, I. hawkeri,* and *I. walleriana*) are widely used as urban ornamental and gardening plants (Jiang et al., 2017; Torrecilha et al., 2013; Kim et al., 2017). *I. balsamina* was also called “zhijiahua” in ancient China, the plant can be mashed and directly applied on the nails (Chen et al., 2007). *I. hawkeri* and *I. walleriana* are annual flowering plants with a high value, they become extremely popular bedding plants (Cafa et al., 2020), and are also used as annual herbs for the treatment of rheumatism, beriberi, bruises, pain, snakebites, fingernail inflammation and onychomycosis in traditional Asian regions (Thakur et al., 2009; Bhaskar, 2012; Szewczyk, 2018). The derivatives of 1,4-naphthoquinones (impatienol and balsaquinone) were proven to be significant in nonsteroidal, anti-inflammatory drug development (Fan et al., 2013; Li et al., 2015). Additionally, previous research has demonstrated that the *Impatiens* species have the potential to accumulate high levels of metals such as copper, zinc, chromium, and nickel (Torrecilha et al., 2013; Lai and Cai, 2016; Campos et al., 2017).

Previous publications have primarily focused on specific geographical regions and divided species into groups by purely descriptive traditional morphology, palynology, and anatomy characters, such as flower, stem, and spur (Yuan et al., 2004; Chen et al., 2007). To date, molecular classification for *Impatiens* was based on morphological characters, several chloroplast plastids (such as coding gene *rbcL, matK, trnK* and intergenic regions *atpB-rbcL* and *trnL-trnF*) (Yuan et al., 2004; Janssens et al., 2006a; Ruchisansakun et al., 2015; Shajitha P. P. et al., 2016). Both of the inter-simple sequence repeat (ISSR) and the nuclear ribosomal ITS markers were utilized in identifying the genetic diversity of populations and the phylogenetic and evolutionary relationships between the *Impatiens* species (Yuan et al., 2004; Shajitha P. P. et al., 2016). The present published data is based on a few samples which only provide regional characteristics with conflicting results, adequate phylogenetic information for an examination of phylogenetic relationships amongst the *Balsaminaceae* species is currently missing (Yu et al., 2016; Li Y. et al., 2018). Sequencing whole chloroplast genomes may remarkably increase the resolution and clarify poorly defined phylogenetic relationships.

The nuclear, chloroplast, and mitochondrial genomes are the three major genetic systems (Yuan et al., 2004; Li ZZ. et al., 2018). Unlike the other genomes, the whole chloroplast genome has a self-replication mechanism, relatively independent evolution, slow evolving nature, and unique maternal inheritance (Park and Lee, 2016; Li et al., 2019). It is feasible for the reconstruction of plant phylogeny and the construction of taxonomy between families and genera from the perspective of population genetics to investigate deep comparisons of angiosperm, gymnosperm, and fern families (Huang et al., 2019). Furthermore, the chloroplast genomes of most land plants are highly conserved in terms of conserved structural regions, size, gene content, and gene types. The conservative and differential gene characteristics can provide vital information for the identification, classification, and phylogenetic reconstruction of relationships among species and families. Chloroplast genomes are also useful in genetic engineering, molecular markers, barcoding identification, and plant evolution (Gu et al., 2018).

Based on medicinal and ornamental values, it is essential to analyze and explore the genetic characteristics of the *Balsaminaceae* species. In the study, we analyzed the chloroplast genome of six phenotypically different species, including three previously published plastid genomes (*I. piufanensis, I. glandulifera,* and *H. triflora*) and three newly sequenced ornamental *Impatiens* species (*I. balsamina, I. hawkeri,* and *I. walleriana*) by using Illumina sequencing technology. The study aimed to: 1) characterize the plastid genome structure of three *Impatiens* species; 2) identify divergence spots among the genomes; 3) reconstruct a plastid genome-based phylogenetic relationships among the available sequences. The present investigation is a novel attempt to reveal and identify the phylogenetic relationship and taxonomic position of the six species based on chloroplast genes. This study will not only contribute to further research on the phylogeny of *Impatiens* species but also provide partly insights into the chloroplast genome evolutionary history of the order *Balsaminaceae*.

## Materials and Methods

### Ethical Statement

No specific permits were required for the collection of specimens for this study. This research was carried out in compliance with the relevant laws of China.

### Materials and DNA Extraction

All leaf samples were collected and identified by Prof. Haiquan Huang, the samples were deposited in the plant Laboratory of the College of Landscape Architecture and Horticulture Science, Southwest Forestry University, Kunming, Yunnan, China (Table 1). The *I. hawkeri* was only sequenced in the previously work, we didn’t analysis it and lack of a well comparion with other species (Luo et al., 2021). Fresh leaves were collected and stored in liquid nitrogen. Total DNA was extracted using the Tiangen DNA Reagent Extraction Kit, and an approximate 5–10 µg of genomic DNA quality was checked (Doyle et al., 1987).

**TABLE 1:** The list of basic information of *Impatiens* species sequenced in this study.

Species	Altitude (m)	Latitude and Longitude	Location	Voucher Specimen
*I. hawkeri*	1953.7	102°76′43″E, 25°06′15″N	Arboretum of Southwest Forestry University, Yunnan Province, China	SWFU-IBXJNY20180811
*I. walleriana*	1953.7	102°76′44″E, 25°06′23″N	Arboretum of Southwest Forestry University, Yunnan Province, China	SWFU-IBSD 20180819
*I. balsamina*	1,094.4	104°71′32″E, 23°12′28″N	Malipo Laoshan Nature Reserve, Wenshan City, Yunnan Province, China	SWFU-IBLH 20180920

### Illumina Sequencing, Assembly, and Annotation

The purified genomic DNA was sequenced by using an Illumina MiSeq sequencer (Biozeron, Shanghai, China) (Bankevich et al., 2012; Langmead and Salzberg, 2012). The clean data were assembled and manually corrected using GetOrganelle version 1.6.2 software (Jin et al., 2018). Each assembled genome was annotated with the GenSeq software (Tillich et al., 2017) and the online Dual Organellar Genome Annotator (DOGMA) (Wyman et al., 2004), the start and stop codon positions were searched by gene identification. The position of tRNAs was confirmed with tRNAscan v1.23 software (Schattner et al., 2005). The notes were manually corrected and verified using Geneious R8.0.2 by realigning with references (Kearse et al., 2012). The reference plastid used is from a closely related species *I. piufanensis* (GenBank MG162586.1). Additionaly, the sequences of the *Balsaminaceae* plants used in this study were downloaded from GenBank as follows: *I. glandulifera* (GenBank MK358447.1), *I. piufanensis*, and *H. triflora* (GenBank MG162585). The online program OGDrawV1.2 generated the circular chloroplast genome maps.

### Repeat Sequence and Simple Sequence Repeats Analysis

The online tool REPuter detected the size and location of repeat types (Kurtz et al., 2017). The Geneious R8.0.2 software was utilized to calculate GC content (Kearse et al., 2012). The online MISA software was used to detect SSRs (Beier et al., 2017). The software CodonW investigated the distribution of codon usage, the distribution of codon usage was investigated with the RSCU ratio (Sharp and Li, 1987).

### Chloroplast Genome Alignment

The multiple alignment of conserved genomic sequence with rearrangements was aligned with the previously published monospecific *H. triflora* chloroplast genome, using the MAUVE software (Darling et al., 2004). MAFFT version was used to detect divergence hotspots (Katoh et al., 2019). The software mVISTA was used to align the whole genome and other species (Brudno et al., 2003; Frazer et al., 2004). The DnaSP v5.10 software was used to calculate the nucleotide divergence values by using the sliding window length of 800 bp and a 200 bp step size (Rozas et al., 2017; R Development Core Team, 2017).

### Phylogenetic Analyses

The MAFFT version 7.222 software was used to align the complete chloroplast genomes with the default parameters (Katoh and Toh, 2010). The Maximum likelihood (ML) and Bayesian Inference (BI) were conducted for the topologies. The ML analysis was implemented in RAxML v.8.2.9. The best-fitting model was a GTR + F + I + G4 substitution with 1,000 bootstrap replicates based on the Akaike information criterion (AIC) (Posada, 2008). The Bayesian inference (BI) tree was implemented in MrBayes version 3.2 (Ronquist et al., 2012). Based on the Markov chain Monte Carlo (MCMC) algorithm, the best-fitting model was a TVM + F + I + G4 substitution within one million generations with four independent heated chains sampled after every 1,000 generations. The FigTree ver1.4.2 software visualized the output trees (Ranbaut. 2014).

## Results

### Features of the Three Ornamental Impatiens

**FIGURE 1:**
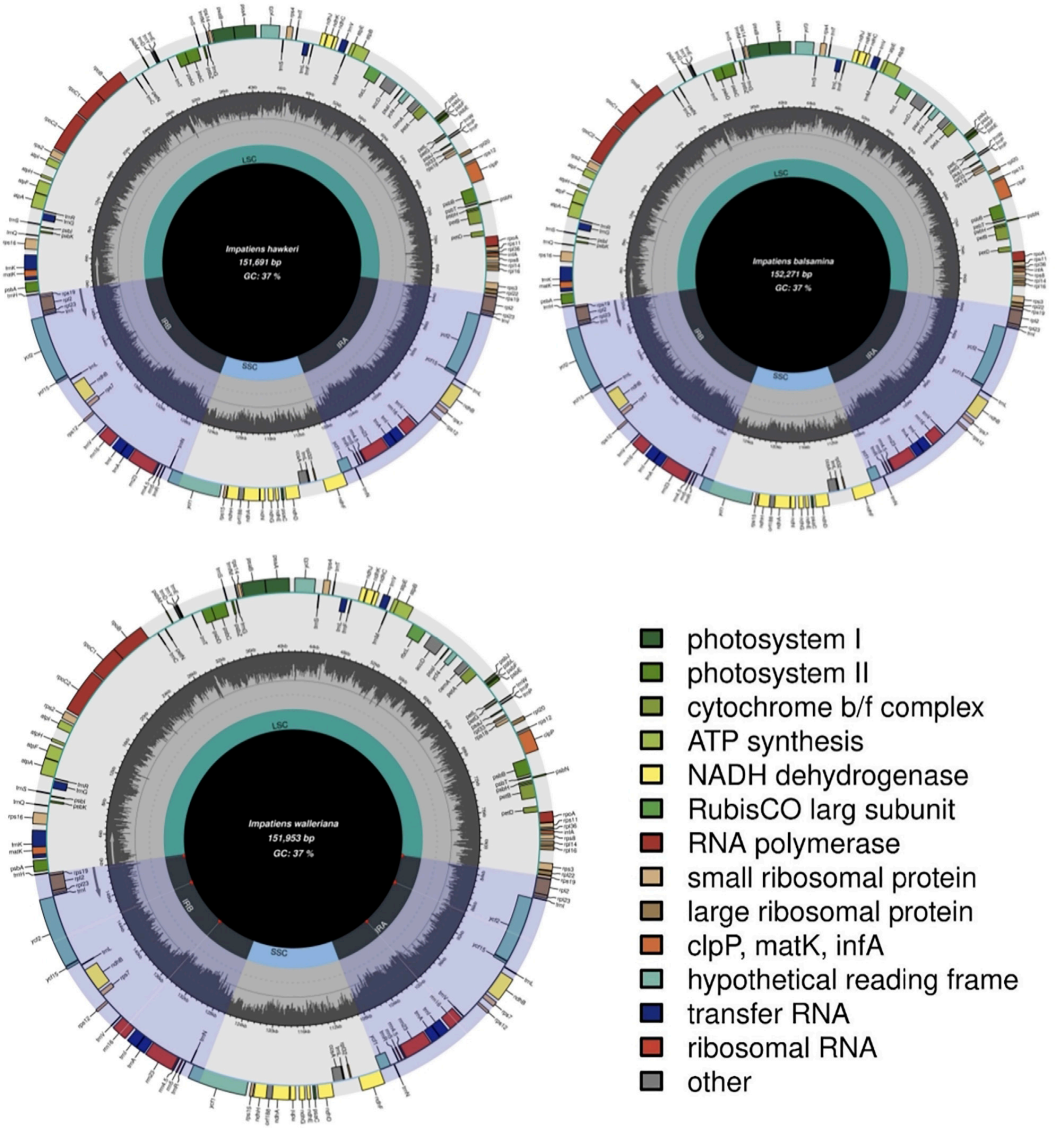
Chloroplast genome structure of three *Impatiens* species (*I. balsamina, I. hawkeri,* and *I. walleriana*).

The total DNA of *I. balsamina, I. hawkeri,* and *I. walleriana* were sequenced using next-generation sequencing technology. As a result, the genomic libraries had a total of 28.6 GB. Contigs mapped to the *I. piufanensis* reference were then used to reconstruct the chloroplast DNA of *Impatiens* where the sizes of *I. balsamina, I. hawkeri,* and *I. walleriana* were 152,271 bp, 151,691 bp, and 151,953 bp, respectively (Table 2 and Supplementary Table S1). The length ranged from 151,691 bp (*I. hawkeri*) to 154,189 bp (*H. triflora*), which consists of a large single copy (LSC, 82,906–83,497 bp), a small single copy (SSC, 17,493–18,276 bp) and a pair of inverted repeats (IRs, 25,249–25,710 bp) (Table 2 and Figure 1). The lengths of *I. hawkeri* and *I. walleriana* were close with *I. balsamina* showing the longest length. The whole guanine-cytosine (GC) contents in the *Balsaminaceae* species ranged from 36.7 to 36.9%, with *I. balsamina* having the lowest and *I. glandulifera* and *H. triflora* having the highest GC content (Table 2). The GC contents in the LSC, IR, and SSC regions were average with 34.4, 43.2, 29.5%, respectively (Supplementary Table S1 and Figure 1).

**TABLE 2:** Characteristics of complete chloroplast genomes for *Impatiens* species.

Species	*I. balsamina*	*I. hawkeri*	*I. walleriana*	*I. piufanensis*	*I. glandulifera*	*H. triflora*
Length/bp	152,271	151,691	151,953	152,236	152,260	154,189
LSC/bp	83,497	83,030	82,906	83,115	83,261	84,865
IR/bp	25,249	25,584	25,710	25,755	25,63	25,622
SSC/bp	18,276	17,493	17,627	17,611	17,737	18,080
Total Genes	114	114	114	114	108	112
CDS	81	81	81	81	80	81
tRNA	30	30	30	30	29	30
rRNA	4	4	4	4	4	4
Total GC content (%)	36.7	36.8	36.8	36.9	36.8	36.9
GC content in LSC/%	34.3	34.4	34.4	34.5	34.5	34.7
GC content in IR/%	43.2	43.2	43.2	43.1	43.1	43.1
GC content in SSC/%	29.3	29.6	29.4	29.3	29.4	29.9

The genetic physical maps of the *I. balsamina, I. hawkeri,* and *I. walleriana* closely resembled the previously published *I. piufanensis*, but the *trnG-UCC* gene was annotated as a pseudogene in *H. triflora* resulting in a total number of 114 genes compared to the other five *Impatiens* species (Figure 1 and Supplementary Figure S1). Another exception is that the genes *ycf15* and *trnfM-CAU* are interchanged due to the incorrect annotation in *I. glandulifera*.

Like other typical angiosperms, the chloroplast genomes of the *Balsaminaceae* species encoded 114 total distinct genes except for *I. glandulifera* and *H. triflora* including 81 protein-coding, 29 transfer RNA genes (tRNA), and 4 ribosomal RNA genes (rRNA) (Table 2 and Supplementary Table S2). Most genes of this genus appear in the form of a single copy in the LSC or SSC region with 20 gene duplications in the IR regions, including *rpl2, ycf1, ndhB, rps7, rps12, rps19, ycf2, rpl23, ycf15*, *trnA-UGC, trnV-GAC, trnI-GAU, trnL-CAA, trnI-CAU, trnR-ACG, trnN-GUU, rrn23 rrn4.5, rrn16,* and *rrn5* (Table 3).

**TABLE 3:** The list of genes in the chloroplast genomes of *Impatiens* species.

Function of genes	Gene groups	Gene names
Photosynthesis-related genes	Rubisco	rbcL
	Photosystem I	psaA psaB psaC psaI psaJ
	Assembly and stability of Photosystem I	ycf3•• ycf4
	Photosystem II	psbA psbB psbC psbD psbE psbF psbH psbI psbJ psbK psbL psbM psbN psbT psbZ
	ATP synthase	atpA atpB atpE atpF• atpH atpI
	Cytochrome b/f complex	petA petB• petD petG petL petN
	Cytochrome c synthesis	ccsA
	NADPH dehydrogenase	ndhA• ndhB•(2) ndhC ndhD ndhE ndhFndhG ndhH ndhI ndhJ ndhK
Transcription and translation-related genes	Transcription	rpoA rpoB rpoC1• rpoC2
	Ribosomal proteins	rpl2•(2) rpl14 rpl16 rpl20 rpl22 rpl23 (2) rpl33 rpl36 rps2 rps3 rps4 rps7 (2) rps8 rps11 rps12•(2) rps14 rps15 rps16•rps18 rps19 (2)
RNA genes	Ribosomal RNA	rrn4.5 rrn5 rrn16 rrn23
	Transfer RNA	trnA-UGC•(2) trnC-GCA trnD-GUC trnE-UUC trnF-GAA trnfM-CAU trnG-GCC• trnG-UCC trnH-GUG trnI-CAU*(2) trnI-GAU•(2) trnK-UUU• trnL-CAA (2) trnL-UAG trnL-UAA• trnM-CAU trnN-GUU(2) trnP-UGG trnQ-UUG trnR-ACG (2) trnR-UCU trnS-GCU trnS-GGA trnS-UGA trnT-GGU trnT-UGU trnV-GAC (2) trnV-UAC• trnW-CCA trnY-GUA
Other genes	RNA processing	matK
	Carbon metabolism	cemA
	Fatty acid synthesis	accD
	Proteolysis	clpP••
Genes of unknown function	Conserved reading frames	*ycf1 ycf2(2) ycf15(2)*

(2) indicates the m = number of the repeat unit is 2; Gene contains one intron; Gene contains two introns.

Introns are missing in the annotations of *I. piufanensis* and *H. triflora*, namely the *trnG-GCC* tRNA gene. 16 unique genes were annotated to include introns, whereas, with 14 genes containing one intron (*rps12, trnI-GAU, trnA-UGC, rpoC1, ndhB, trnK-UUU, trnG-GCC, ndhA, rpl2, petB, atpF, rps16, trnv-UAC,* and *trnI-UAA*); and the *ycf3* and *clpP* genes each containing two introns (Table 3 and Supplementary Table S3). The *rpoC1* gene had the longest exon and the *rps12* gene had the longest intron.

### Codon Usage

To analyze the genetic information and the relationship between evolution and phylogeny of *Impatiens*, we examined the codons in its coding region. The total number of codons was 304,804. The significant number of codons identified in the different species was as follows: 50,757 (*I. balsamina*), 50,503 (*I. hawkeri*), 50,651 (*I. walleriana*), 50,745 (*I. piufanensis*), 50,753 (*I. glandulifera*), and 51,395(*H. triflora*) (Supplementary Table S4). Among the 20 AAs, the most abundant AA was leucine (29,142, 9.56%), followed by isoleucine (25,482, 8.36%). Tryptophan had the lowest frequency AA in the *Balsaminaceae* species and was encoded by only 3,960 codons (1.2%). Among species, codon usage based on the relative synonymous codon usage value (RSCU) had not changed, except for some reductions found in five AAs of *I. piufanensis, I. glandulifera, I. balsamina, I. hawkeri,* and *I. walleriana. H. triflora* had 36 codons which were more frequently used than the expected usage at equilibrium (RSCU>1). *I. glandulifera* had 30 codons which were less frequently used than the expected usage at equilibrium (RSCU<1).

### Repeat Structure and Simple Sequence Repeats Analyses

**FIGURE 2:**
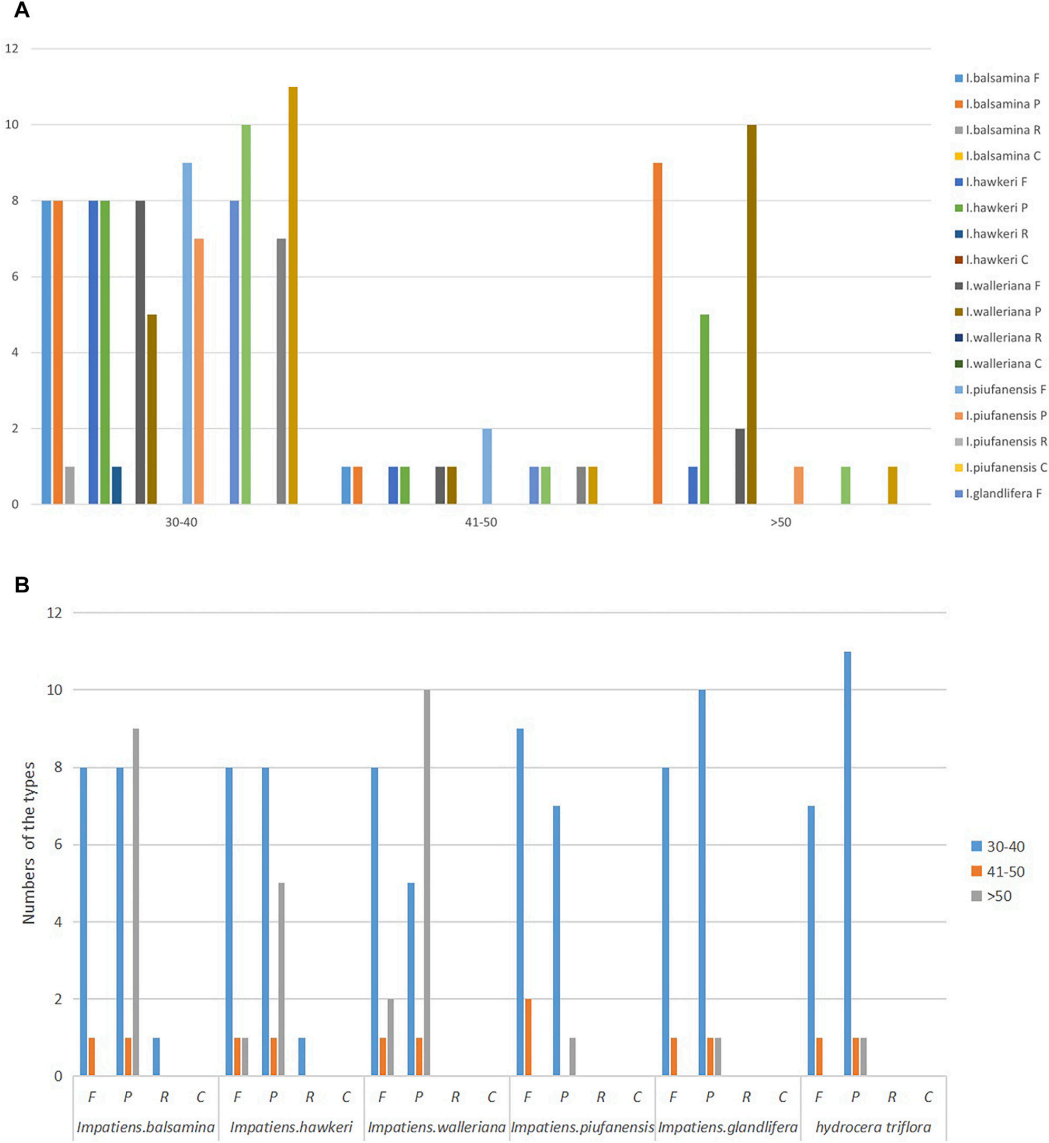
Analysis of repeated sequences in the *I. balsamina, I. hawkeri, I. walleriana, I. piufanensis, I. glandulifera,* and *H. triflora* chloroplast genomes. **(A)** A total of six species of four repeat types by length; **(B)** Total six species of four repeat types.

A total of 141 unique forward, complement, reverse, and palindromic repeats were examined among the six *Balsaminaceae* species using REPuter software. *I. balsamina* contained a total of 28 repeats including 18 palindromic repeats, 9 forward repeats, and 1 reverse repeat (Figure 2). In *I. hawkeri, I. walleriana, I. piufanensis, I. glandulifera,* and *H. triflora*, 24, 22, 18, 20, and 20 total repeat pairs were detected, respectively (Supplementary Table S5). Among all six species, the most common repeat types were palindromic and forward repeats, compliment repeats were not identified, and reverse repeats were only found in the *I. balsamina* and *I. hawkeri* species, respectively. Most of the repeat lengths were less than 40 bp, however, the *I. balsamina* and *I. hawkeri* chloroplasts had 2 forward or palindromic repeats with a length of between 41 and 50 bp.

**FIGURE 3:**
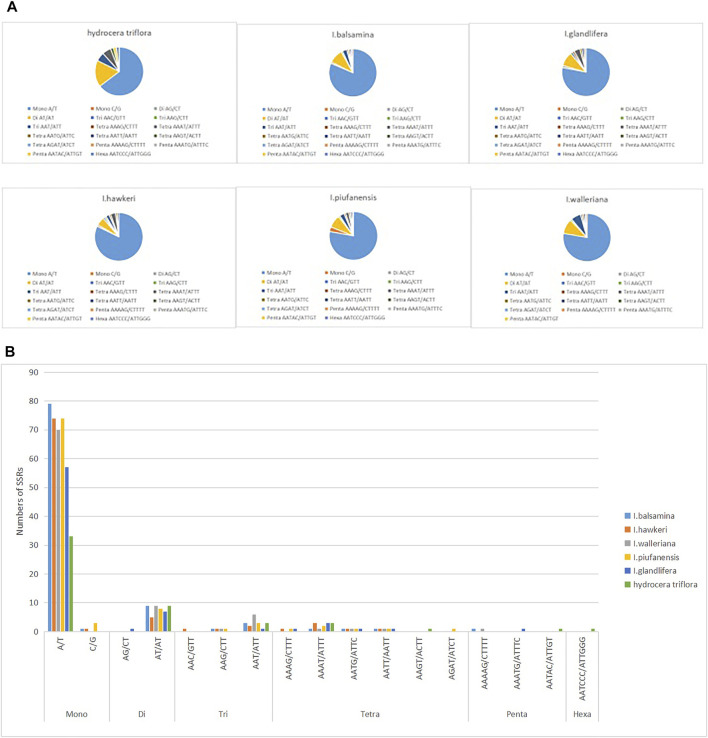
Analysis of simple sequence repeats (SSRs) in the chloroplast genomes of *I. balsamina, I. hawkeri, I. walleriana, I. piufanensis, I. glandulifera,* and *H. triflora*. **(A)** The number of different SSR types detected in each species; **(B)** type and frequency of each identified SSR.

**FIGURE 4:**
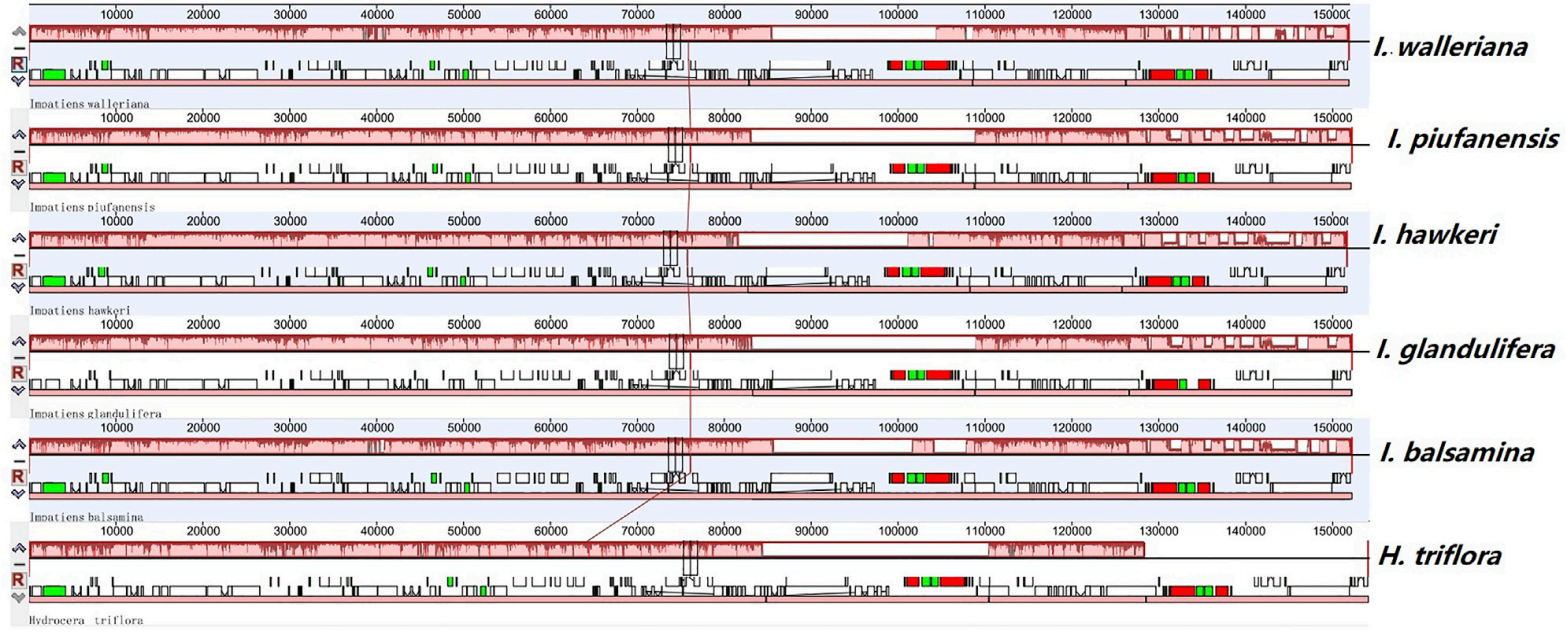
Comparison of sequence arrangement in the chloroplast genomes of six *Balsaminaceae* species.

Among the six *Balsaminaceae* species, there were 97, 90, 91, 95, 96, and 51 SSRs in the *I. balsamina, I. hawkeri, I. walleriana, I. piufanensis, I. glandulifera,* and *H. triflora* chloroplast genomes, respectively (Figure 3 and Supplementary Table S6). Mononucleotide repeats were more abundant with A/T repeats being the most highly represented repeats with a size of 33–79, which accounted for about 64.7–81.44% of the total SSRs, while poly C/G repeats were rather rare (0–3.15%). Among the dinucleotide repeat motifs, AT/AT were the most abundant, while AG/CT only found in *I. glandulifera*. Three trinucleotide motifs (AAC/GTT, AAG/GTT, AAT/ATT), six tetranucleotide (AAAT/ATTT, AAGT/ACTT, AATG/ATTC, AATT/AATT, AAAG/CTTT), three pentanucleotide (AATAC/ATTGT, AAAAG/CTTTT, AATAG/ATTCT) were identified (Figure 4). However, only one hexanucleotide (AATCCC/ATTGGG) repeat was found in the *H. triflora*.

### Comparison of the Genome Structure in *Balsaminaceae*

Most chloroplast genomes in angiosperm plants are relatively stable. However, based on different evolutionary histories and genetic backgrounds, the chloroplast genome structure, size, and numbers can vary. Collinear blocks were used to analyze and compare the collinearity of chloroplast genomes. The mauve alignment for the six *Balsaminaceae* species revealed that the optimal collinearity within subgenus *Impatiens* is relatively conserved and lacks gene rearrangement (Figure 4). Compared with *H. triflora*, the linear relationships within genome structure and gene sequences indicated that there was high chloroplast genome homology.

### Inverted Repeat Expansion and Contraction

**FIGURE 5:**
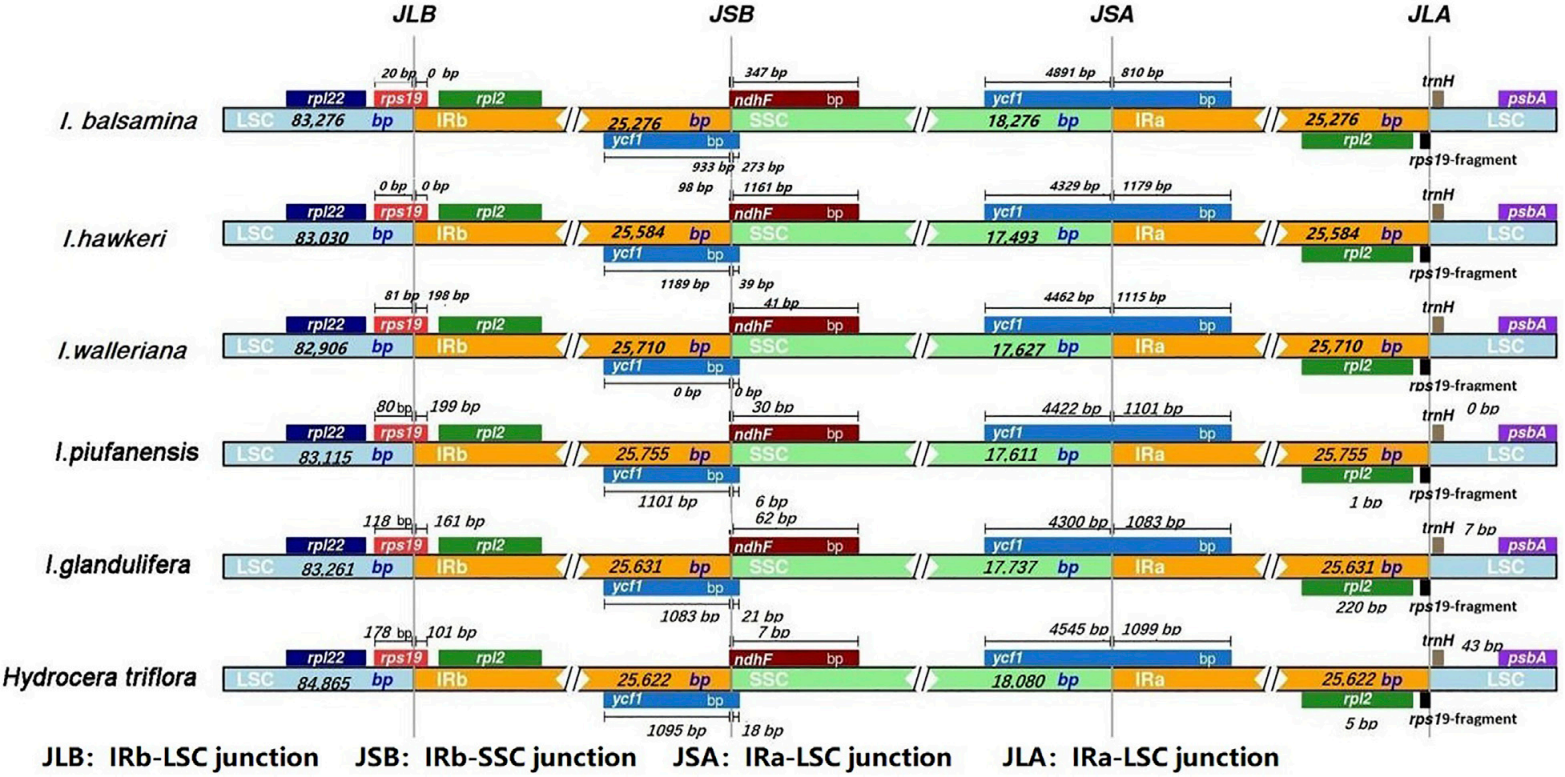
Comparison of the borders of four different regions (LSC, SSC, and IRs) among *I. balsamina, I. hawkeri, I. walleriana, I. piufanensis, I. glandulifera,* and *H. triflora* chloroplast genomes.

Four junctions in regions of detailed structure were compared among the *Balsaminaceae* and subsequently presented (Figure 5). The IRb-LSC junction (JLB) was located in the *rps19* coding region which was inserted between the IRB and LSC region in all six species. The length of the *rps19* in the IRB region among the four species (*I. walleriana, I. piufanensis, I. glandlifera,* and *H. triflora*) had varied from 101 to 199 bp. Notably, the length of the *rps19* in the IRB region of both *I. balsamina* and *I. hawkeri* was 0 bp. The SSC-IRB junction (JSB) was adjacent to gene *rps19* and *ndhF*; JSB of six species except for *I. walleriana* were all located and adjoined the end of *ycf1* from 933 bp to 1,189 bp. The overlap between *ndhF* and *ycf1* was detected in *I. hawkeri*, with *ndhF* expanding into the IRB region for 1,161 bp. In the other five species, the distances between *ndhF* and JSB were 347, 41, 30, 62, and 7 bp, respectively. The IRA-SSC junction (JSA) was located in the *ycf1* coding region which covered the IRA and SSC region. The length of *ycf1* in the SSC region varied from 4,300 bp to 4,545 bp. However, six species overlap *ycf1* in the IRA region were found 810, 1,179, 1,115, 1,101, 1,083, and 1,099 bp, respectively. The LSC-IRA junctions (JLA) were located between *rpl12* and *rps19* in *I. balsamina* and *I. hawkeri*, while in other four species, the distances between *traH* and *rpl12* were 0 bp, 0 bp, 7 bp, 43 bp, respectively. In the JLA junction, the *rps19* gene was 34 bp and 104 bp into the LSC region in *I. balsamina* and *I. hawkeri,* while the distances between *rpl2* and JLA were 25, 46, 1, 1, 220, and 5 bp, respectively.

### Comparative Genomic Divergence and Genome Rearrangement

**FIGURE 6:**
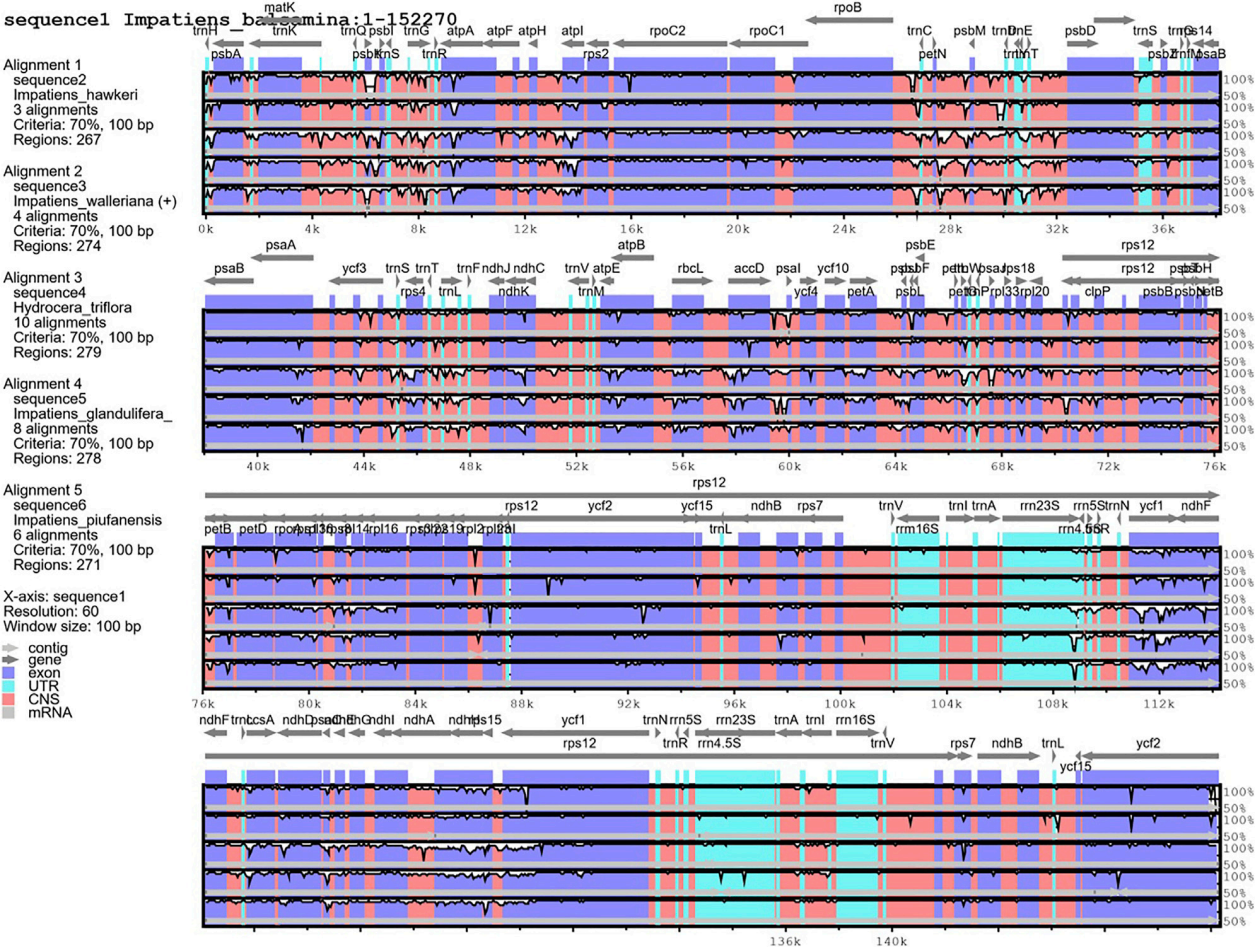
Alignment of the six chloroplast genomes. Sequence identity plot comparing the five chloroplast genomes with *I. balsamina* as a reference by using mVISTA.

The mVISTA program was used to detect hyper-variable regions based on whole regions of chloroplast genomes. *H. triflora* and other *Impatiens* species showed sequence divergence in many regions such as *rps3-rps19, matK, psbK, atpH-atpI, trnC-trnT, petN, psbM, atpE, rbcL, accD, psaL, ycf1, ndhG-ndhA,rpl16, rpoB, ndhB, ndhF,* and *ndhH* (Figure 6). The three genes; *ndhF, ycf1,* and *ndhH* were detected in the SSC region. The *psbK-psbI, atpI*, and *rps4-trnF* genes showed some divergence in the LSC region of *I. piufanensis, I. glandlifera,* and *H. triflora.*

**FIGURE 7:**
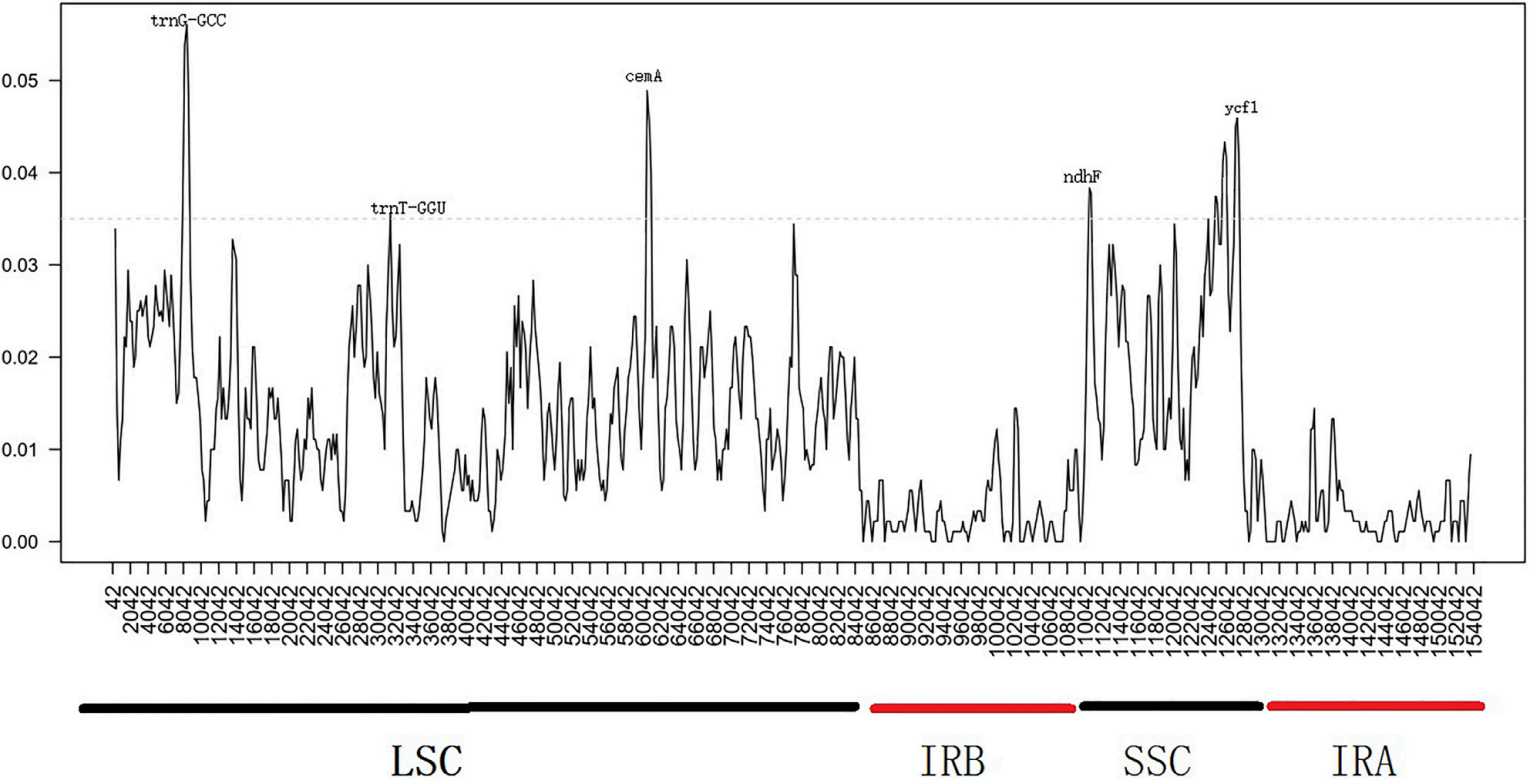
Sliding window analysis based on the chloroplast genomes of three *Balsaminaceae* species. Window length: 2000 bp; step size: 200 bp. X-axis: the position of the midpoint of a window. Y-axis: nucleotide diversity of each window.

Similarly, we determined the average pairwise sequence divergence among three ornamental species of *Impatiens* chloroplast genomes. The nucleotide variability (Pi) of these 140 regions ranged from 0.1% (*ycf2*) to 5.6% (*trnG-GCC*) among three chloroplast genomes (Supplementary Table S7). Additionaly, ten different genes; *psbA, trnS-trnG, trnG-GCC, atpH-atpL, trnE-trnT, psbD, cemA, ndhF, rpl32, ndhA,* and *ycf1* were sequenced within these genomes. The *trnG-GCC* gene demonstrated the highest average sequence divergence (0.056), followed by *cemA* (0.048), and *ycf1* (0.046) (Figure 7). Sliding window analysis indicated that mutational hotspots included *psbA, trnS-trnG, trnG-GCC, atpH-atpL, trnE-trnT, psbD,* and *cemA*, which exhibited higher Pi values (>0.035) in the LSC and SSC regions. Single mutational hotspots in the IR regions with remarkably high PI values (>0.015) were not present.

### Phylogenetic Analysis

**FIGURE 8:**
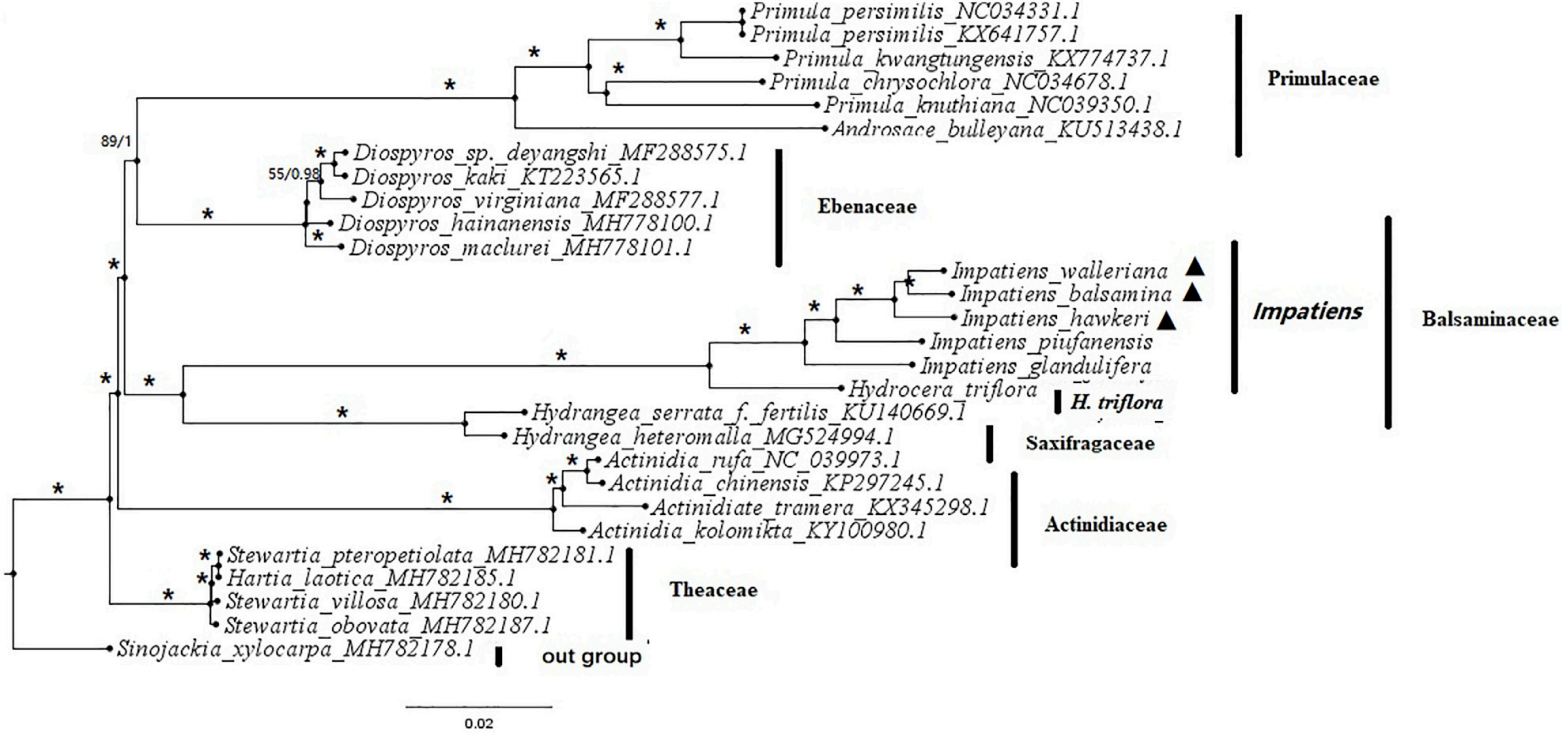
Phylogenetic tree based on whole chloroplast genome sequences from 6 *Balsaminaceae* species and 23 other species using maximum likelihood (ML) bootstraps and Bayesian posterior probabilities (PP). ML topology is shown with ML bootstrap support values/Bayesian PP given at each node. Asterisks indicate both of PP = 1 and LBS = 100%. Black triangles indicate the cp genomes of the three Impatiens species examined in this study.

An exploration of the phylogenetic positions and evolutionary relationships of *Impatiens* species based on the complete chloroplast genomes (Supplementary Table S8). The chloroplast genomes from seven families within six *Balsaminaceae* species, six Primulaceae species, five Ebenaceae species, four Theaceae species, two Saxifragaceae species, four Actinidiaceae species, and one Styracaceae species as outgroup. The topologies of the two datasets (ML and BI) yielded a similar structure. The seven families can be classified into five monophyletic clades (Figure 8). Actinidiaceae was the basal group in all phylogenetic trees. The Primulaceae and Ebenaceae were gathered into one clade and also the *Balsaminaceae* was a sister to Saxifragaceae. Most of the species from the same genus were clustered together. All *Balsaminaceae* species formed a monophyletic subclade in both trees. *H. triflora* was located at the bottom of the *Balsaminaceae* phylogenetic tree and clustered into a single clade. All Impatiens species were clustered into one clade, The cultivated species; *I. balsamina, I. hawkeri,* and *I. walleriana* were more closely related than the wild species *I. piufanensis* and *I. glandulifera*.

## Discussion

### Genome Structure

Compared with the reported genome structures among *Balsaminaceae*, the family was slightly smaller in size with 151,691 bp (*I. hawkeri*) of the former to 154,189 bp (*H. triflora*) of the latter (Table 2 and Supplementary Table S1). There was a 2,498 bp difference in length between the *Balsaminaceae* species. Nevertheless, the basic structure and content of the genome were roughly similar (Yu et al., 2016; Li Y. et al., 2018). Chloroplast genomes were found to be highly conserved. The potential of *ycf15, trnfM-CAU,* and *psbN* genes had been annotated in all genomes of *Impatiens* species, while in *H. triflora* they were not excluded in this study. Likewise, the reading frames named the *trnG-UCC* gene which had been only annotated in *I. glandulifera*. Based on observations, their ability to encode proteins in angiosperms has not yet been confirmed. The results indicate homology in genome structure, therefore, that may be decisively resolves the systemic evolutionary relationship for species identification and taxonomy. The genes were divided into three categories based on function (Tanner et al., 2014). The first was related to photosynthesis and translation genes, such as Rubisco, ATP synthase, Cytochrome b/f complex, assembly, and stability of Photosystem I, II (Tamboli et al., 2018). The second category corresponds to Ribosomal and Transfer RNA (Beerling and Perrins, 1993); and the third category contained biosynthetic genes, such as Carbon metabolism gene *cemA,* Proteolysis gene *clpP,* fatty acid synthesis gene *accD,* and some unknown function genes (*orf188, ycf1, ycf2,* and *ycf15*) (Hulme and Bremner, 2006).

### Inverted Repeat Expansion or Contraction

By detecting detailed boundary changes of the regions, we observed that the IR-SC boundary regions showed minimal differences (Figure 5). Some extensions or contractions were detected, with the IR regions ranging from 25,276 bp to 25,755 bp (in *I. balsamina* to *I. piufanensis,* respectively)*.* Variations of *rps19, ycf1, ndhF,* and *rpl2* genes were observed and partially duplicated genes were found at the beginnings and ends of the IR regions including 178 bp of *rps19* in *H. triflora,* and the *rps19* gene of *I. hawkeri* not extending into the IR region. The SSC and LSC regions showed higher sequence divergence than the IR regions. Moreover, the pairwise alignment of the *I. balsamina* showed high synteny with other species. Similarly, most divergent genes were detected, especially in *psbA, trnS-trnG, trnG-GCC, atpH-atpL, trnE-trnT, psbD, cemA, ndhF, rpl32, ndhA,* and *ycf1* (Figure 7). The coding regions in all *Balsaminaceae* chloroplast genomes showed less divergence than the non-coding regions. As previously reported, *trnG-GCC, cemA,* and *ycf1* genes possessed high variability as possible molecular markers. Therefore, these coding regions and non-coding genes may provide strong molecular evidence for resolving low-level phylogeny and phylogeography (Fujihashi et al., 2002; Li et al., 2015).

### Repetitive Sequence Analyses

Based on the analysis of various chloroplast genomes, repetitive sequences were essential for inducing indels and substitutions (Zuo et al., 2017; Yan et al., 2019). The sequences not only play a vital role in the rearrangement and stabilization of the chloroplast genome sequence but also affect the copy number differences between similar and different species (Xie et al., 2018; Wang et al., 2020). The *Impatiens* chloroplast genome had four different repetitive sequences. The forward repeats can be used as markers in phylogenetic studies due to the changes in genomic structure. Among all species, the most common type of repetition was a palindrome repeat. All species contained forward and palindromic repeats but compliment repeats were not identified in all species while reverse repeats were only found in *I. balsamina* and *I. hawkeri* (Figure 2 and Supplementary Table S5).

Simple sequence repeats (SSRs) have been recognized as a marker for having a high polymorphism rate and abundant variation at the species level (Wang et al., 2020). Moreover, SSRs can be used to detect genetic diversity, population, and polymorphisms at intraspecific, distant phylogenetic relationships and cultivar levels. Our analysis identified the distribution of 51–97 SSRs in the *Balsaminaceae* species ranging from 10 to 20 bp in size (Figure 3 and Supplementary Table S6). Furthermore, not all the SSR types were identified in all the species, hexanucleotide and pentanucleotide repeats were not detected in *I. hawkeri* and *I. pinfanensis,* while the hexanucleotide repeats were found only in *H. triflora.*

### Phylogenomic Validation

Analysis of the whole chloroplast genome can effectively solve the various problems in molecular evolution and the phylogeny of the same genus or family, hence it can enhance our understanding of molecular evolution (Janssens et al., 2009; Shajitha P. P. et al., 2016). The first molecular phylogeny of the genus was published by Fujihashi. However, due to limited taxon sampling and the use of a distant outgroup *Tropaeolum* (Tropaeolaceae), findings were limited information on the systemic evolutionary relationships (Fujihashi et al., 2002). Nuclear ribosomal internal transcribed spacer (ITS) and *atpB-rbcL* sequences for studying on 111 *Balsaminaceae* species, provided new phylogenetic insights, namely that the *Impatiens* had colonized from Southwest China to the African continent in three separate proliferation events (Janssens et al., 2006b; Shajitha P. P. et al., 2016). Subsequently, plastids, plastids and nuclear, or combined plastids and pollen data collected from the *Impatiens* were further analyzed (Yuan et al., 2004). A new classification of *Impatiens* based on morphological and molecular datasets divided them into two subgenera: *Clavicarpa* and *Impatiens* with *Impatiens* being further subdivided into seven sections based on morphological characteristics or combinations of the ITS results, *atpB-rbcL,* and *trnL-F* intergenic fragments, along with pollen data (Yu et al., 2016). Although the new schematic provided a robust basis for further research, all the published data contained only a few samples from obvious regional samples and the results were conflicted.

In the present study, based on the maximum likelihood (ML) and Bayesian Inference (BI) trees (Figure 8). Two phylogenetic trees showed the same results. The three selected families (Actinidiaceae, Theaceae, and Styracaceae) were clustered into a monophyletic branch, respectively. The Genus *Primula* and *Androsace* of the family Primulaceae were clustered into a clade, the family Theaceae also consisted of the *Stewartia* and the *Hartia Dunn*. The *Balsaminaceae* and Saxifragaceae were clustered into a clade. All *Balsaminaceae* species formed a subclade in both ML and BI trees. And *H. triflora* and *Impatiens* formed two different subclades (Figure 8). The *I. balsamina, I. hawkeri,* and *I. walleriana* species with the most similar morphological characteristics were clustered together, suggesting highly consistent phylogenetic relationships in morphology and genomics, and also were very likely to be derived from one species, and had the same ancestor (Yuan et al., 2004; Rahelivololona et al., 2018). The species *I. piufanensis* and *I. glandulifera* were closer to *H. triflora* in the *Balsaminaceae*, which may have experienced the same habitat and evolutionary process.

Similarly, the results of the similarities and differences identified the phylogenetic relationships between the Impatiens species by sequencing whole chloroplast genomes, traditional morphology and molecular classification indicated that the phylogenetic trees from the three cultivars of *I. hawkeri, I. walleriana,* and *I. balsamina* were in a relatively unique evolutionary position. Compared with the wild species, the cultivated species had a very high bootstrap value and an obvious evolutionary trend. Based on previous phylogenetic analyses using the ITS and matK fragments, the phylogenetic trees were divided into different clades (Yuan et al., 2014; Tamboli et al., 2018). In terms of morphology, except for *I. balsamina*, which is an annual herb, the other two were perennials (Chen, 2001); the stem was fleshy and the leaves of *I. hawkeri* were whorled and the other two were alternate, stalked (Yu, 2012; Yu et al., 2016); *I. walleriana* had ovate leaves, with the other two species having lanceolate leaves with sharp teeth on the edge (Chen et al., 2007; Yu, 2012); The three cultivars had the same morphology: solitary flowers without pedicels; two pieces lateral sepals; obliquely ovoid, round flag petals with keel-like protrusions, wing petals with short stalks, lip petals; boat-shaped; anthers spherical; fusiform ovary, and capsule fusiform (Cai et al., 2013). However, using the BI and ML morphology and molecular phylogenetic trees can be well integrated.

The resulting phylogenomic tree highly supported the clade of the *Balsaminaceae* species forming a monophyletic subclade, with the clusters of cultivated and wild species, confirming the validity of the assembled and annotated chloroplast genome of *Balsaminaceae* species, which is consistent with the results of plastid genes and supports the classification of Ericicales in the updated APG IV system (Janssens et al., 2009; Li ZZ. et al., 2018). The use of chloroplast genome data clearly reflects the evolutionary relationship between wild *impatiens* and cultivated species, and decisively resolves the systemic evolutionary relationship between wild species and cultivated *Impatiens*. The research shows that we have clearly identified the phylogenetic and taxonomic position of the three cultivated species in the *Impatiens* genus, and provides molecular evidence that the chloroplast genome can be applied to clarify phylogenetic questions within or between the *Impatiens* genus. The comparative analyses using whole chloroplast genomes provided an important new perspective into genome structure and resolved multiple inconsistencies in molecular evolution and genus phylogenetic relationships.

## Conclusion

Three different ornamental species (*I. balsamina, I. hawkeri,* and *I. walleriana*) and three novel wild species of the genus *Impatiens* were analyzed in this study. They proved to be valuable genomic resources in the present examination of the *Balsaminaceae* family. The results showed a highly similar basic structure, size, GC content, gene number, order, and functional array. Similarly, most divergent genes were detected, mutational regions contained highly variable nucleotide hotspots that may be used as potential markers for species identification and taxonomy. Additionally, based on the ML and BI phylogenomic trees, the trees highly supported three different ornamental species forming a monophyletic subclade. The comparative analyses using whole chloroplast genomes provided an important new perspective into genome structure and resolved multiple inconsistencies in molecular evolution and genus phylogenetic relationships. However, the Impatiens consists of approximately 1,000 species, which makes it complicated to identify species by determining the whole genome of chloroplast. Future research on *Balsaminaceae* relationships needs a larger sampling of taxa, morphological characteristics combined with simple molecular markers, and genome-wide analyses to enhance our understanding of evolution.

## Data Availability

The datasets presented in this study can be found in online repositories. The names of the repository/repositories and accession number(s) can be found in the article/Supplementary Material.

## Abbreviations

BI, Bayesian Inference; bp, base pairs; Gb, Gigabases; IGR, Intergenic region; IR, Inverted repeat; ITS, Internal transcribed spacer; LSC, Long single copy; LSR, Long sequence repeat; MCMC, Markov chain Monte Carlo; ML, Maximum likelihood; NCBI, National Center for Biotechnology Information; NGS, Next-generation sequencing; PCR, Polymerase chain reaction; PI, Parsimony informative; rRNA, ribosomal RNA; SSC, Short single copy; SSR, Simple sequence repeat; tRNA, transfer RNA.
