# Supplementary material for: Complete Chloroplast Genomes and Comparative Analyses of Three Ornamental Impatiens Species
Source: Front Genet. 2022 Mar 30;13:816123. doi: 10.3389/fgene.2022.816123 (PMC9006450; doi:10.3389/fgene.2022.816123)

| **Figure S1 Chloroplast genome structure of three Impatiens species (*I. balsamina I. hawkeri,* and *I. walleriana*)** |
| --- |


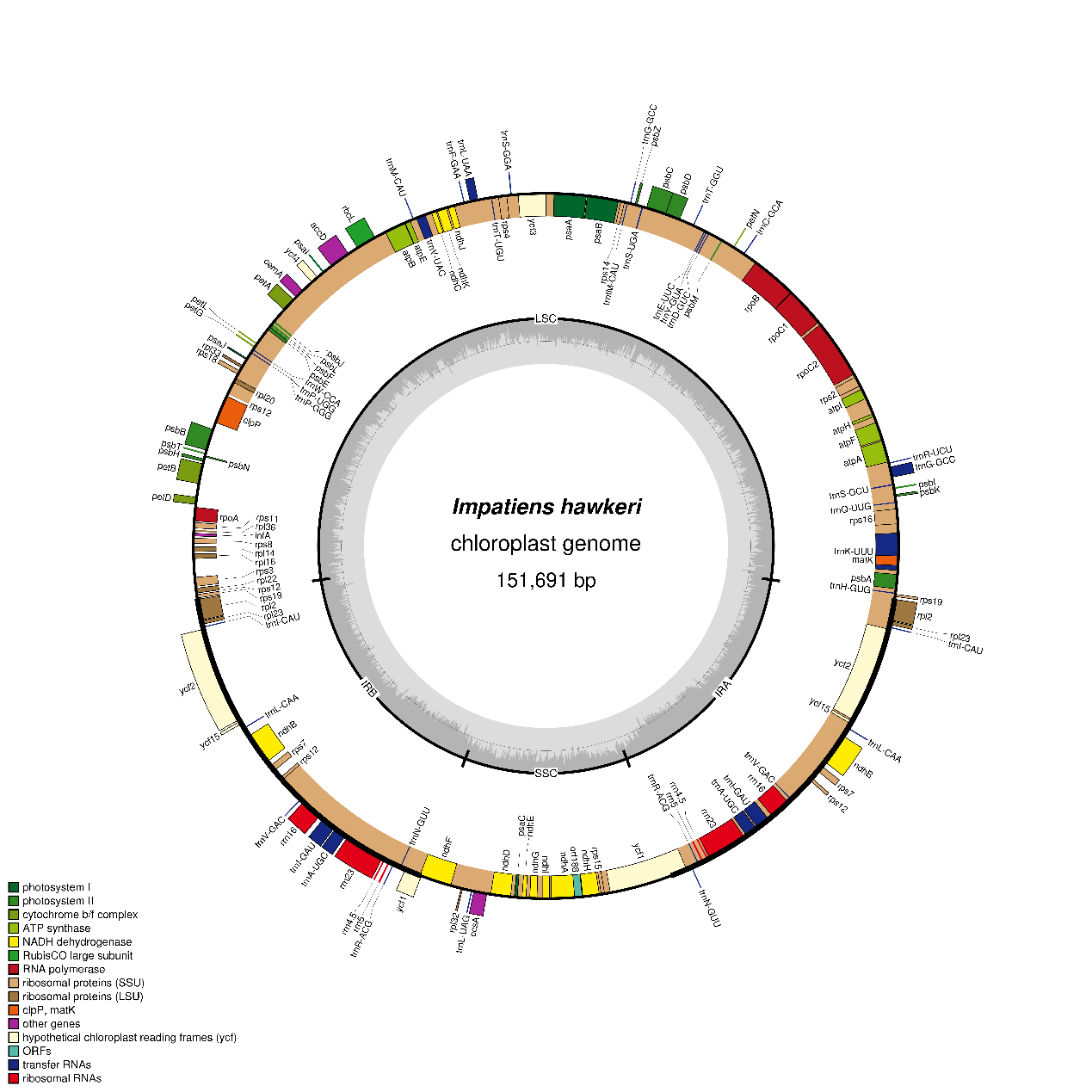


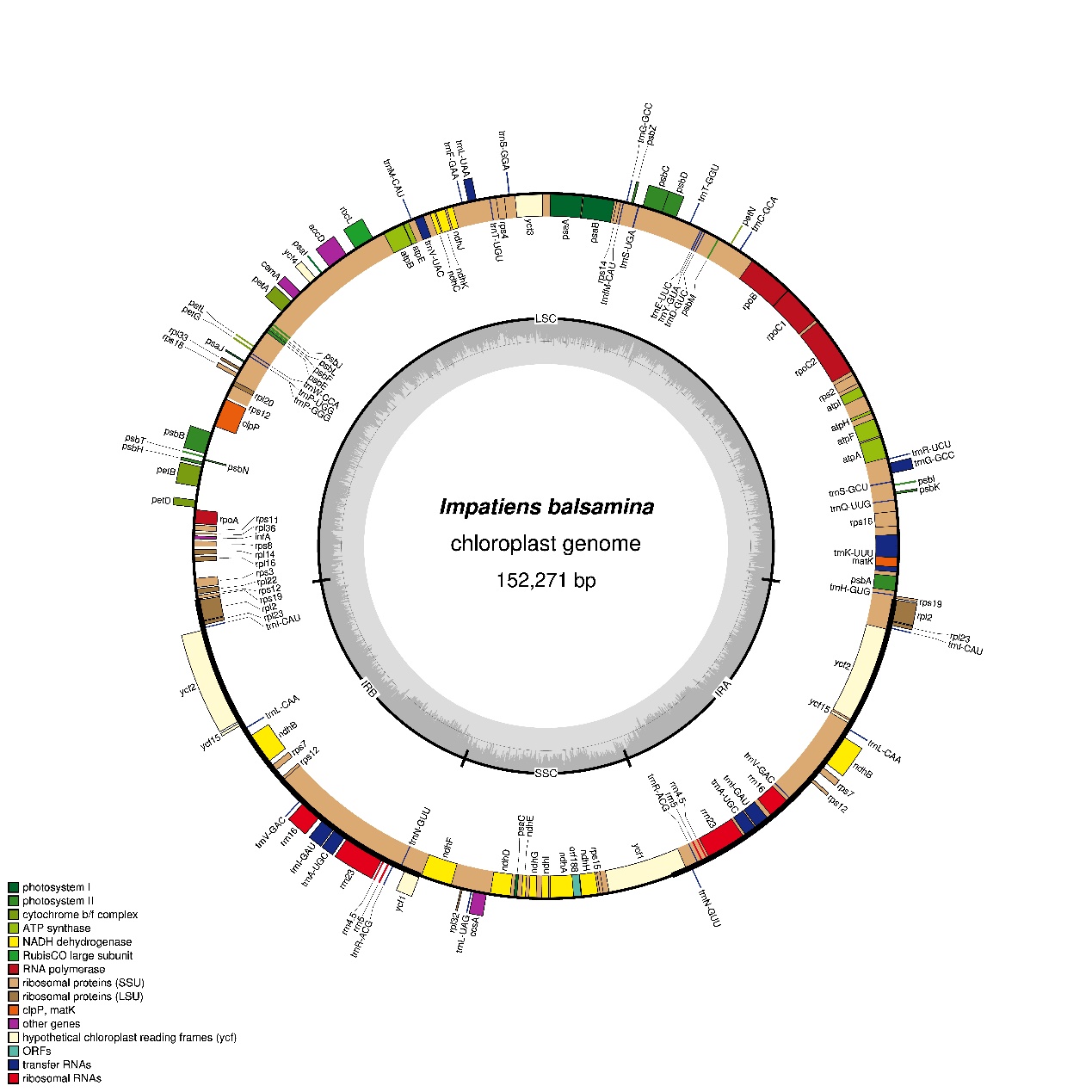


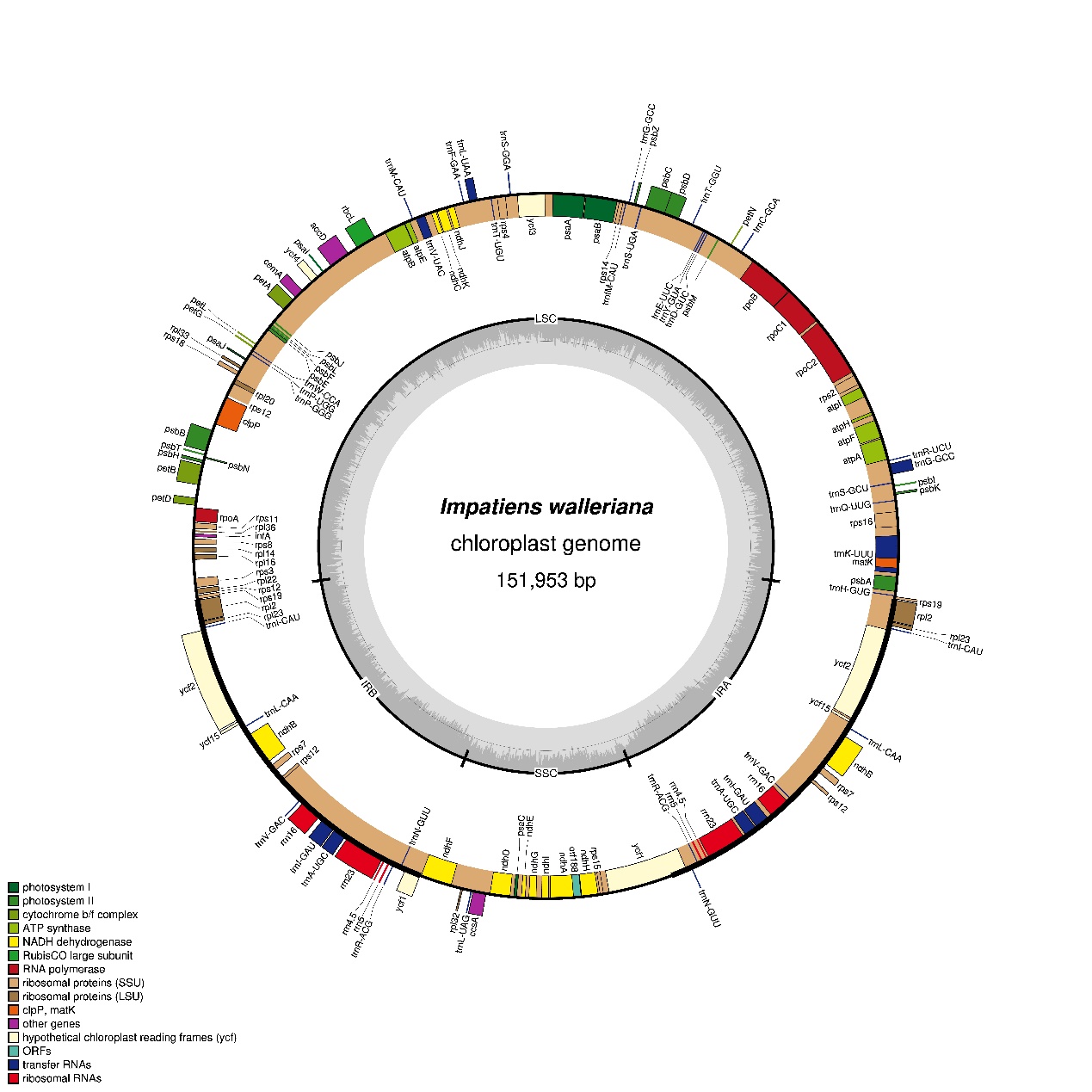

Supplement: Supplementary file 1 [file DataSheet1.ZIP › Supplementary/Supplementary Figure 1.DOCX]
